# Is there a risk of permanent renal dysfunction after primary total hip and knee joint replacements?

**DOI:** 10.1186/s13018-016-0457-z

**Published:** 2016-10-19

**Authors:** Basim Kamil Hassan, Ram Benny Christian Dessau, Arne Sahlström

**Affiliations:** 1 Department of Orthopedics, Nykoebing Falster Hospital, Fjordvej 15, 4800 Nykoebing Falster, Denmark; 2 Department of Clinical Microbiology, Slagelse Hospital, Faelledvej 1, 4200 Slagelse, Denmark

**Keywords:** Knee, Hip, Permanent renal dysfunction, Joint arthroplasty

## Abstract

**Background:**

Permanent renal dysfunction is considered as being a serious complication which may occur after major surgery and which furthermore may lead to increased morbidity and mortality. The objective of this study was to analyze the incidence of long-term postoperative renal dysfunction after primary total hip and knee joint replacements.

**Methods:**

Long-term postoperative renal dysfunction was analyzed in a retrospective study of 1301 consecutive primary total hip and knee joint replacements performed between January 2009 and December 2013. According to the RIFLE criteria, increased serum creatinine was an indicative of postoperative renal injury. The highest serum creatinine during the first postoperative week was chosen as a sign for maximum acute renal injury and was compared to the highest serum creatinine during the following 4–12 months.

**Results:**

One hundred and forty two patients with an increase in postoperative serum creatinine were included in the follow-up study. Six patients (4.2 %) died due to non-renal causes during the follow-up period. One patient died of severe renal injury, which was relatively very early postoperatively, and another patient had a rise in serum creatinine to 316 μmol/l during the follow-up period. All the remaining 132 patients (94 %) had full recovery with serum creatinine which returned to preoperative levels.

**Conclusions:**

This study did not confirm that patients who underwent primary total hip and knee joint replacement surgery were at risk of developing permanent renal dysfunction up to 1 year after the index surgery.

## Background

Renal injury and impairment have been reported in a few studies after primary total hip and knee joint replacements [1–5]. To the authors’ knowledge, there has been only one publication regarding the permanent renal dysfunction after a subgroup of primary hip joint replacements [6]. Previous publications have dealt with short-term postoperative renal dysfunction [2, 7, 8] but not with permanent renal dysfunction after major surgery. The aim of this study was to analyze the incidence of long-term postoperative renal injury after total hip and knee joint replacements.

## Methods

Long-term postoperative renal dysfunction was analyzed in a retrospective study of 1301 primary consecutive total joint replacements; 702 primary knee joint arthroplasties were performed between January 2009 and December 2012, and 599 primary hip joint arthroplasties were performed between January 2011 and December 2013. The group of cases described in the present study was a subset of cases described in two previous studies [4, 5]. The primary total joint replacements were all performed in our department by joint-replacement surgeons for primary osteoarthritis (1184), posttraumatic osteoarthritis (57), rheumatoid arthritis (13), and femoral neck fractures (48). The data was collected from our computerized database and hospital charts. There was one or more missing pieces of data for 66 patients, and thus, they were excluded from the primary studies [4, 5]. However, they did not differ from the investigated patients included in the study regarding age, gender, American Society of Anesthesiologists (ASA) score, body mass index (BMI), hypertension, diabetes mellitus, smoking, duration of surgery, type of anesthesia, prophylactic antibiotic, and blood pressure (baseline and intraoperative systolic and diastolic BP) (Fig. 2). Non-steroidal anti-inflammatory drugs (NSAID) were not used in the postoperative management for patients included in the study.

The highest serum creatinine during the first postoperative week was chosen as a sign for maximum acute renal injury and used to calculate the RIFLE score [9, 10]. This was compared to the highest serum creatinine measurement during the following 4–12 months (Table 1). All patients were routinely followed up for assessment of knee and hip function 2 months postoperatively. In addition, patients with moderate or severe postoperative renal dysfunction have had contact with the hospital as part of their follow-up for renal function-related issues or for other diseases in which blood tests were taken including serum creatinine.

**Table 1:** Characterization of 142 cases with a rise in serum creatinine postoperatively

Item	Distribution
Age in years at operation, median (range)	76 (38–94)
Sex, number (% female)	85 (60 %)
BMI, median (range)	28 (18–42)
Smoking	28 (20 %)
Diabetes mellitus	17 (12 %)
ASA score at the operation: 1, 2, or 3	12 (8 %), 95 (67 %), 35 (25 %)
Hypertension	104 (73 %)
Mortality	7 (5 %)
Diagnosis: osteoarthritis, fracture, others	128 (90 %), 10 (7 %), 4 (3 %)

As a control group, we randomly checked serum creatinine for a period of 4–12 months postoperatively in 111 patients from the group of patients who have had none or very mild postoperative renal dysfunction. The randomization method depended on choosing the patients with social security number closest to the probands. This control group did not have any increase in serum creatinine for a period of 4–12 months postoperatively.

All calculations and graphics were performed in R statistical software (http://www.r-project.org/). Preoperative serum creatinine was compared to serum creatinine at follow-up using a paired *t* test.

## Results

**Fig. 1:**
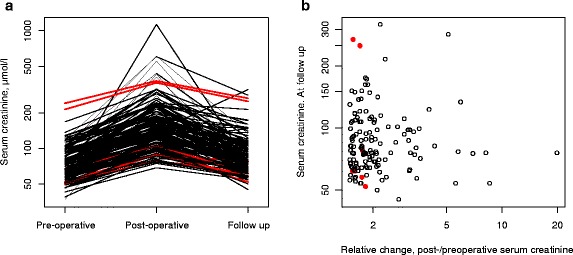
Serum creatinine of 142 patients. **a** Interaction plot showing preoperative, postoperative, and follow-up at 4–12 months. **b** *xy*-plot of serum creatinine at follow-up as a function of relative perioperative change. *Red color* shows cases with mortality in the follow-up period. *Numerical axes* are scaled as the natural logarithm

All 142 patients (10.9 %) who had significant acute renal injury according to RIFLE classification (RIFLE ≥1.5) [9, 10] were followed up for a period of 4–12 months postoperatively until serum creatinine returned to normal or worsened to the development of permanent renal injury. A minimum of one measurement of serum creatinine was obtained under the follow-up period on all 142 patients except for one patient (0.7 %) who died from severe renal failure relatively early postoperatively. Six patients (4.2 %) died during the follow-up period as a result of non-renal causes. One patient had a rise in serum creatinine to 316 μmol/l during the follow-up period. All the remaining 132 patients (94 %) had full recovery with serum creatinine returning to preoperative levels without further permanent renal dysfunction. Figure 1a illustrates the pattern of serum creatinine in 142 patients starting preoperatively, peaking within 1 week of surgery, and returning in 94 % of patients to preoperative levels. Figure 1b illustrates no correlation of serum creatinine at follow-up compared to perioperative change in serum creatinine. The control group of 111 did not have any increase in serum creatinine 4–12 months postoperatively.

## Discussion

**Fig. 2:**
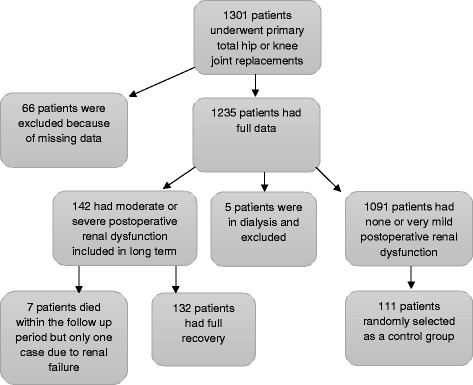
Flow chart showing the number of patients included in the study and their follow-up

Chronic or permanent renal injury is important regarding morbidity and mortality in any given population. This study could not confirm a risk of permanent renal injury in 1301 patients who underwent primary total hip or knee joint replacements save for one patient who died because of renal failure, which was probably due to major surgery, but other comorbidities could have played a role (Fig. 2).

Chandran and Giori [6] identified in a study involving a 9-year follow-up an increased risk (14 %) of chronic renal disease after primary metal-metal hip joint replacement in a population which was extracted from the Department of Veterans Affairs VistA database. However, the health status in the study group differed from the general population in that they had more medical conditions and a higher medical resource use compared with the general population as showed by Agha et al. in his study of 128,099 records from the National Health Interview Survey for the years 1993 and 1994 [11].

The RIFLE classification [9, 10] has been used in several studies in respect to the postoperative acute renal injury [1–5, 7, 8], but to our knowledge, it has never been used to assess long-term chronic postoperative renal injury. This study could not confirm that the RIFLE classification has a role as a useful predictor of chronic postoperative renal injury.

## Conclusions

This study could not confirm that the primary total hip and knee joint replacements might induce permanent renal dysfunction up to 1 year after the index surgery. The use of the RIFLE classification is not recommended as a reliable predictor of permanent renal dysfunction after surgery.

## Abbreviations

ASA: American Society of Anesthesiologists
BMI: Body mass index
BP: Blood pressure
NSAID: Non-steroidal anti-inflammatory drugs
RIFLE: Risk, injury, failure, loss of function, end-stage renal disease
